# Erratum

**DOI:** 10.4269/ajtmh.916err

**Published:** 2014-12-03

In *Am J Trop Med Hyg 88:* 48 by Fairley et al., there is an error in the percentage of low birth weight (LBW) in the study population. The incorrect value currently listed is 15.4%. This was inadvertently calculated using the incorrect definition of ≤ 2.5 kg. Using the universally accepted definition of LBW is < 2.5 kg, the true percentage was 9.5%. There were several infants with weights right at 2.5 kg, hence the discrepancy. However, all of the analyses used the correct definition of < 2.5 kg, so the error lies solely in the descriptive statistics of the study population.

